# A Web-Disseminated Self-Help and Peer Support Program Could Fill Gaps in Mental Health Care: Lessons From a Consumer Survey

**DOI:** 10.2196/mental.4751

**Published:** 2017-01-19

**Authors:** Samantha L Bernecker, Kaitlin Banschback, Gennarina D Santorelli, Michael J Constantino

**Affiliations:** ^1^ Department of Psychological and Brain Sciences University of Massachusetts Amherst, MA United States; ^2^ Division of Education Queens College City University of New York Flushing, NY United States

**Keywords:** computer-assisted instruction, eHealth, mental health, self care, self-help, peer support, social support

## Abstract

**Background:**

Self-guided mental health interventions that are disseminated via the Web have the potential to circumvent barriers to treatment and improve public mental health. However, self-guided interventions often fail to attract consumers and suffer from user nonadherence. Uptake of novel interventions could be improved by consulting consumers from the beginning of the development process in order to assess their interest and their preferences. Interventions can then be tailored using this feedback to optimize appeal.

**Objective:**

The aim of our study was to determine the level of public interest in a new mental health intervention that incorporates elements of self-help and peer counseling and that is disseminated via a Web-based training course; to identify predictors of interest in the program; and to identify consumer preferences for features of Web-based courses and peer support programs.

**Methods:**

We surveyed consumers via Amazon’s Mechanical Turk to estimate interest in the self-help and peer support program. We assessed associations between demographic and clinical characteristics and interest in the program, and we obtained feedback on desired features of the program.

**Results:**

Overall, 63.9% (378/592) of respondents said that they would try the program; interest was lower but still substantial among those who were not willing or able to access traditional mental health services. Female gender, lower income, and openness to using psychotherapy were the most consistent predictors of interest in the program. The majority of respondents, although not all, preferred romantic partners or close friends as peer counselors and would be most likely to access the program if the training course were accessed on a stand-alone website. In general, respondents valued training in active listening skills.

**Conclusions:**

In light of the apparent public interest in this program, Web-disseminated self-help and peer support interventions have enormous potential to fill gaps in mental health care. The results of this survey can be used to inform the design of such interventions.

## Introduction

### The Need to Consult Consumers When Developing Web-Based Mental Health Interventions

Self-guided, Web-based health interventions have great promise in reaching consumers and improving public health. Web-based *mental health* self-help treatments may have even greater advantages in reach over traditional in-person treatments because they afford more self-reliance and privacy and they minimize stigma, circumventing common barriers to seeking mental health help from a professional [1].

However, self-guided, Web-based interventions come with their own challenge: they need to be appealing enough to motivate consumer engagement. Internet-based interventions often report low user adherence, with many users discontinuing the intervention prematurely [2-4]. On the basis of a diffusion of innovations framework, Eysenbach [2] has suggested that one factor influencing attrition rates may be “the degree to which an innovation is perceived as being consistent with the existing values, past experiences, and needs of potential adopters.” In response to the low uptake of health interventions, many researchers and organizations have begun to call for an iterative development cycle in which consumers are consulted repeatedly in order to ensure that interventions are appealing to them and meet their needs [5-7].

### Our Intervention: “Crowdsourcing Mental Health”

In response to these calls, our research team sought consumer input on a new intervention that is called “Crowdsourcing Mental Health” (CMH) because it distributes the task of providing mental health care among the general public. In CMH, 2 individuals who are already acquainted with each other (eg, friends, coworkers) agree to participate in the program as “partners.” Both partners take an asynchronous massive open online course (MOOC) that teaches 2 sets of skills: skills for talking about stressors and skills for listening to others who are stressed. After completing the course, the partners meet weekly for peer counseling, taking turns in the “talker” and “listener” roles. This reciprocal structure allows both partners to gain the benefits of care provision [8] and care receipt, as well as to develop the intimacy that results from mutual self-disclosure [9].

Stress can precipitate the onset of, or worsen, a variety of mental illnesses (eg, [10-12]), and it has negative consequences for physical and mental health regardless of one’s diagnostic status [13,14]. In order to help users manage stress, the CMH program teaches coping skills that are based on the literature on adaptive and maladaptive coping processes (eg, [15,16]). Like stress, disrupted emotion regulation has been linked to mental illness (eg, [17,18]). To improve emotion regulation, the CMH course includes psychoeducation about emotion regulation and teaches identification and labeling of emotions, which may promote adaptive responding [19-21]. Finally, the CMH program leverages the power of interpersonal relationships, both to improve well-being and to promote adherence and engagement. By “talking through” the steps of coping and emotion regulation with a supportive peer rather than on one’s own, the CMH program is intended to enhance intimacy and strengthen perceived social support, which has well-established links to mental and physical health [22-24].

### This Study

CMH and programs like it have the potential to improve public mental health in principle, but the success of such programs depends upon consumer interest and user adherence. In accordance with recommendations that consumer consultation be the first step in developing public health interventions [6,25], we conducted a survey to investigate the public interest in CMH and assess consumer preferences for specific elements of Web-based self-help and peer support programs, questions that remain unanswered in the existing literature (as discussed below). Addressing these questions would allow us to predict whether CMH was a viable strategy for improving public mental health and to tailor CMH to consumer preferences in order to optimize its appeal, effectiveness, and reach.

The specific research questions for this investigation were as follows. Although we had tentative hypotheses for some of these questions, this study should be viewed as exploratory, not confirmatory.

#### Research Question 1: Overall Interest

Is consumer interest adequate to justify investment in development of the CMH program and interventions like it?

A major goal of the survey was to gauge general public desire to participate in a program like CMH to estimate the potential reach of this program. Deciding upon a precise threshold for “adequate” interest would require knowledge of the cost-effectiveness of this program, which does not yet exist. However, we decided a priori that it would unquestionably be worthwhile to pursue the development of CMH if 30% of participants endorsed willingness to try the program. This was a conservatively high bar: even if the survey overestimated the proportion of users by 90%, this would mean about 10 million persons in the United States would try the program.

#### Research Question 2: Interest Among Those Not Accessing Traditional Services

Is consumer interest high enough among those who are not accessing or who do not plan to access traditional mental health care, and how does this group’s level of interest compare with that of people who do access traditional care?

Common barriers to seeking mental health help from a professional include structural or practical impediments such as cost, inconvenience, and provider unavailability, as well as attitudinal barriers such as feeling that one’s problem is not severe enough to warrant professional help or wanting to avoid stigma [1]. Because it is freely available on the Web, does not require interacting with a mental health professional, and is not presented as a treatment for mental illness, CMH has the potential to circumvent these barriers, so we hoped that some proportion of those who would not access traditional care would endorse willingness to try CMH. Indeed, eHealth and mHealth research suggests that technology-based interventions can reach those who have been underserved by traditional health care [26,27]. Nevertheless, CMH shares some characteristics with traditional psychotherapy (eg, taking time each week to open up about one’s problems). Because of these similarities, we hypothesized that those who were not willing to access traditional care would be less interested in CMH than those who were willing to access traditional care. We decided a priori that a 20% endorsement of willingness to try CMH within this group would be adequate to justify development of CMH.

#### Research Question 3: Characteristics Associated With Interest

What user characteristics are associated with interest in the program?

Characterizing the potential audience is a key step in tailoring health interventions [25]. Identifying possible “early adopters” and nonusers can inform strategies for tailoring or advertising Web-based mental health interventions to enhance their appeal to specific populations [27].

Some research has been conducted on predictors of attitudes toward mental health care or eHealth, but findings have not been consistent, and they may not apply to the nontraditional CMH program because it is intended to have broader appeal than traditional mental health services. Thus, further investigation is needed.

On the basis of current literature, one might expect females to be more interested than males [28-30], people of Asian descent to be less interested than those who are white [31-33], people with higher income [29,33,34] and more education [29,35,36] to be more interested, married or cohabiting people to be more interested than those who are not partnered [33,34,36], and past mental health care users to be more interested than those who have not accessed mental health care [29,33,37]. Findings regarding age differences in willingness to seek mental health care have been mixed [29,33,37,38], but age is inversely associated with use of eHealth [28], so one might expect that older people would be less interested in CMH because it will be disseminated via the Internet. We did not have specific expectations about the other characteristics investigated, such as psychological distress, because of contradictions or null findings in past research (eg, [33,37,39,40]).

#### Research Question 4: Preferred Partners

Whom would prospective users most want to have as a partner?

Most existing peer support programs have paired individuals with strangers, but to our knowledge there is no empirical justification for doing so. In fact, pairing users with individuals with whom they are already acquainted may have advantages, such as eliciting more disclosure [41], making meetings more convenient, and improving those relationships. On the other hand, users might rather not work with individuals with whom they are already close because they want to keep personal matters private from those people, because their close relationships are conflictual, or because they want the opportunity to develop a new relationship. Thus, research on consumer perspectives is necessary to differentiate among these rival possibilities.

#### Research Question 5: Desired Skills

Which peer counseling skills would prospective users want their partners to learn? Do these skills differ from the skills they would want to learn themselves?

We also inquired about which counseling skills people wanted to learn most, as well as which counseling skills they want their partner to learn. Delivery of appropriate social support is notoriously fraught, and the support provided is often suboptimal, mismatching the situation or the recipient’s characteristics (eg, [42-44]). Perceived social support is optimized by matching the recipient’s desires and goals, not the provider’s [45,46]. Therefore, research is needed to clarify the type of support potential users would like to receive in CMH (ie, what skills they want their partners to learn) in order to determine what skills to teach. Investigating discrepancies between desired support provision and receipt will elucidate mismatches in what support providers want to deliver and what recipients actually want to receive; this information can then be used to educate CMH users about what types of supportive behaviors to avoid. We had no specific hypotheses about which skills would be most preferred, but we expected that, for many skills, there would be significant discrepancies between desire to learn the skill oneself versus desire for the partner to learn the skill (in light of the aforementioned potential for incongruence between support providers’ and recipients’ goals).

#### Research Question 6: Preferred Access Channels

How do 6 possible access channels compare in likelihood of use, appeal, trustworthiness, convenience, and ease of use?

Finally, we investigated the ideal access channel, offering several options for the Web-based platform and the venues through which users would learn about the program. Respondents ranked 6 options (website, app, social media, physician, community center, and school or work), then rated each channel on attributes that have been shown to predict technology and service adoption: appeal [47], ease of use [47,48], convenience [49], and trustworthiness [48,50].

Although there is evidence that these attributes are important, there is surprisingly little academic research available regarding consumer perspectives on channels of eHealth access (although it is likely that individual organizations and market research firms have collected proprietary data on related questions). With regard to Web-based platforms, we expected that respondents would report being more likely to use a stand-alone website than a mobile app or social networking site, given that a website can be accessed on a wide variety of devices; the percentage of US adults who own a laptop or desktop computer (73%) is slightly higher than those who own a smartphone (68%; [51]) and those who use social networking (65%; 76% of Internet users; [52]). We also expected that a stand-alone website would be viewed as more trustworthy than a mobile app or social networking given previous reports of user security concerns about the latter two platforms [53,54]. However, we had no hypotheses about the other attributes for these platforms or the relative merits of different organizations through which users could be alerted to the existence of health technologies.

## Methods

### Recruitment

Survey respondents were drawn from a convenience sample of potential health care consumers: users of Amazon’s Mechanical Turk (MTurk), a work crowdsourcing website where any individual older than 18 years can complete simple tasks, including psychological experiments, for pay [55]. Although MTurk workers are not perfectly representative of the general public, they are far more demographically comparable to the US population than other Web or college student samples, and results obtained with MTurk samples are comparable to those obtained via other methods [56]. MTurk users also display rates of mental illness similar to or slightly greater than the general population [57]. Therefore, MTurk users should provide a useful approximation of potential users of our peer counseling intervention. The survey was administered in March 2014.

### Eligibility

Access to the survey was limited to MTurk users with Internet protocol (IP) addresses in the United States. Users with the same IP address were prevented from completing the survey to avoid duplicate entries. We required MTurk users to have at minimum a 95% approval rate and at least 50 prior tasks completed on the MTurk website in order to access the survey.

For their data to be included in the analyses, participants need to correctly answer 3 simple comprehension questions after reading a description of the program. Because these questions were extremely straightforward (eg, “What do you learn from the training: How to speak another language, how to be helpful when listening and talking to your partner, or how to improve your memory?”), incorrect responses were indicative of inattention or gross misunderstanding of the description, rendering respondents’ reactions to the program invalid.

### Survey Content

Because the majority of survey content was unique to this project and therefore had not been validated in prior research, we pilot-tested and refined the survey through cognitive interviewing [58] to enhance validity. In 6 cognitive interviews conducted by the first or second author, pilot test participants completed the survey while describing their thought processes out loud and responded to a set of interviewer prompts assessing their interpretations of survey content. Survey items were altered or removed when the interviews revealed that the items were ambiguous, were not applicable to some respondents, or elicited interpretations that differed from their intended meaning.

#### Program Description

The survey began with a description of the program accompanied by stick figure illustrations. The description explained that, in the program, 2 people who were already acquainted would take a Web-based course in scientifically supported talking and listening skills, then would meet weekly in person to put the skills to use, and it listed a variety of potential benefits of participating. The complete description appears in Multimedia Appendix 1.

#### Interest in Program

Interest in the program was assessed in 3 ways. Respondents indicated intention to try the program on a dichotomous yes or no item (“Would you try the program?”) and a 4-option forced-choice item. Additionally, participants responded to 12 items measuring attitudes toward the program (eg, “This program could help solve a problem I have”), including 5 reverse-scored items (eg, “This program would be a waste of my time”), rated on a 7-point Likert scale with anchors from “strongly agree” to “strongly disagree.” The mean of the Likert-rated scale items was computed to create a composite continuous attitude score. This attitude scale was internally consistent (coefficient alpha=.94). Finally, we gave respondents the opportunity to provide their email addresses in order to receive more information about the program with the assumption that this, as a behavioral indicator of interest, required more commitment than a verbal claim of hypothetical willingness to try the program.

#### Desired Program Features

The survey also included items assessing desired features of the program. Participants were asked to rate their interest in learning 11 social support skills and in having an imagined partner learn the same skills. These skills were selected from those that, according to qualitative studies of social support, are frequently delivered by well-intentioned support providers, although they may or may not be perceived as helpful by support recipients [59-61].

Survey respondents also ranked 5 possible types of people they would prefer to have as a partner, 5 possible channels through which to meet with their partner, and 6 possible ways to access the course. They rated their perceptions of the possible ways to access the course on 4 semantic differentials: *appealing* to *unappealing*, *trustworthy* to *untrustworthy*, *convenient* to *inconvenient*, and, for the technological channels only, *easy to use* to *hard to use*.

#### Personal Information

Demographic characteristics and information on mental health service use were collected using straightforward, study-specific items. Rather than using total household income in subsequent analyses, we corrected for household size by dividing household income by the square root of the number of people in the household, an adjustment that assumes some economy of scale within the household, such that each additional household member costs the household less than the previous one [62].

Psychological distress was assessed using the Brief Symptom Inventory (BSI) [63], a 53-item instrument that is a shortened form of the Symptom Checklist-90-Revised. Participants rate symptoms experienced within the past week on 9 mental illness dimensions (Depression, Anxiety, Obsessive-Compulsive, Hostility, Somatization, Interpersonal Sensitivity, Paranoid Ideation, Psychoticism, and Phobic Anxiety), from which an index of total distress, the Global Severity Index, can be calculated. The Global Severity Index typically has excellent internal consistency (coefficient alpha>.90 [64]) and test-retest reliability of .90 over a 2-week interval [63]. The BSI displays both convergent validity with other measures of psychopathology and predictive validity in correctly classifying individuals as patients [63,64]. In our sample, coefficient alpha was .97.

All surveys were approved by the University of Massachusetts Amherst Institutional Review Board and were carried out in accordance with all applicable regulations. Before consenting, participants were informed about the investigator’s identity; the purpose, length, and risks and benefits of taking part in each survey; and the methods of data storage.

### Statistical Analysis

We addressed missing data by using the package mice [65] for the software R (R Core Team) to generate and pool 5 multiply imputed datasets. This package implements Rubin’s [66] rules for pooling, with Barnard and Rubin’s [67] approximation for degrees of freedom. The fit of nested models is compared using the methods described by Meng and Rubin [68].

To identify demographic and clinical predictors of interest in the program, we conducted a series of regressions for each of the 3 outcome variables: attitude score (continuous), willingness to try the program (dichotomous), and provision of an email address (dichotomous). In the first step of these exploratory analyses, predictors were entered hierarchically in blocks. This allowed for tests of the incremental predictive effect of a group of related variables; for example, all race or ethnicity dummy codes were entered in a block, enabling a test of whether race or ethnicity significantly improved model fit, which would not have been possible if all variables were entered simultaneously. We expected that some of the predictors entered in the same block would be collinear (eg, education and income), but would act as indices of the same construct (eg, socioeconomic status or SES), and we would interpret the test of whether that block improved model fit as an indicator of whether the overall construct predicted interest in the program. The order of the blocks was based on what Cohen and colleagues [69] refer to as “causal priority.” That is, variables are entered earlier when they logically cannot be caused by other variables in the model, whereas variables entered later could be caused by earlier variables. For example, gender may cause level of psychological distress, but psychological distress cannot cause gender. In cases when reciprocal causation was possible (eg, SES and psychological distress), we entered the variable that is usually evident earlier in an individual’s life course first.

After conducting these hierarchical regressions with all variables, we then repeated the procedure, omitting any blocks of predictors that did not marginally improve model fit (*P*<.10). We then ran a final simultaneous regression in which we removed any individual predictors that did not approach significance when entered in order in the previous series of regressions (*P*<.10) in order to obtain estimates of the effect of each variable while controlling for all other relevant variables (including those for which reciprocal causation was possible). Retaining “marginally significant” covariates in the final model (ie, using a cutoff of *P*<.10 as opposed to *P*<.05) constitutes a more conservative test of the independent predictive ability of each variable, while eliminating those that did not approach significance in earlier models reduces noise and increases parsimony.

To compare participants’ interest in their partners versus themselves learning each support skill, we conducted paired *t* tests for each skill.

To identify whether specific potential partners, meeting channels, or course access methods were ranked significantly higher than others, we compared each pair of possible choices with a Wilcoxon signed rank test, with a Bonferroni correction for multiple comparisons.

## Results

### Characteristics of the Sample

Of the 637 MTurk users who completed the survey, 592 (92.9%) correctly answered all 3 comprehension questions and were therefore eligible for inclusion. Missing data were relatively rare; all 592 individuals responded to the majority of items such that there were no missing values for any attitude items, mental health and distress items, gender, race, marital status, or education; the maximum number of missing values for any variable was 8 (for income).

Demographic, clinical, and service use characteristics of the sample are presented in Table 1. Of note, respondents evidenced a wide range of symptom distress, with approximately a quarter scoring above the BSI’s suggested clinical cutoff (ie, their symptom severity was consistent with the presence of mental illness).

**Table 1:** Participant characteristics.

Characteristics		Mean (SD) or n (%) (N=592)
Age in years, mean (SD)		37.37 (13.11)
**Gender, n (%)**		
	Male	212 (35.8)
	Female	375 (63.3)
	Gender nonconforming (eg, transgender, genderqueer)	5 (0.8)
**Race or ethnicity, n (%)**		
	White, non-Hispanic	458 (77.4)
	Black	44 (7.4)
	Hispanic	33 (5.6)
	Asian	27 (4.6)
	Native American	6 (1.0)
	Other race or ethnicity	24 (4.0)
**Marital status, n (%)**		
	Married or cohabiting	275 (46.5)
	Never married	256 (43.2)
	Separated or divorced	50 (8.4)
	Widowed	11 (1.9)
**Education, n (%)**		
	High school or less	64 (10.8)
	2-Year degree	51 (8.6)
	Some college	177 (29.9)
	4-Year degree	184 (31.1)
	Some graduate or professional school	31 (5.2)
	Graduate or professional degree	85 (14.4)
**Income in US $, mean (SD)**		
	Median household income	42K
	Income per person^1/2^	32.9K (22.7K)
**Brief Symptom Inventory**		
	Score, mean (SD)	0.5045 (0.5985)
	In “clinical” range, n (%)	142 (24.0)
**Psychotherapy use, n (%)**		
	Currently in therapy	44 (7.4)
	Ever in therapy	261 (44.1)
	Would consider trying therapy	403 (68.1)
**Psychiatric medication use, n (%)**		
	Currently prescribed medication	73 (12.3)
	Ever prescribed medication	175 (29.6)
	Would consider trying medication	362 (61.1)

### Overall Interest in the Program

More than half the respondents (378/592, 63.9%; 95% CI 60.0% to 67.7%) indicated on the dichotomous item that they would try the program. When asked to choose from 4 options, 14.9% (88/592) indicated that they “would sign up now,” 46.5% (275/592) indicated that they “might try it in the future,” 29.2% (173/592) indicated that they “would probably not try it,” and 9.5% (56/592) indicated that they “would never try it.” For the behavioral indicator of interest, approximately one-third of the respondents (193/592, 32.6%; 95% CI 28.8% to 37.8%) volunteered their email addresses in order to request more information about the program. The median score on the continuous attitude scale was 5.1 out of a possible range of 1 to 7 (mean 4.9, SD 1.1), corresponding to an anchor of “somewhat agree” on positively worded items.

To assess whether our program would appeal to those who lacked access to or interest in traditional mental health interventions, we computed the percentages of those who had not used psychotherapy or psychiatric medication but who would try our program, as well as the percentages of those who stated that they would not be willing to use those traditional interventions but who would try our program. Of those who never accessed psychotherapy, 62.5% indicated that they would try the program, compared with 65.5% of those who had used therapy, χ^2^_1_=0.4, *P*=.51. Of those who said that they would not be willing to use therapy if they had a problem, 51.3% indicated they would try our program, while 69.7% of those who were willing to use therapy would try our program, χ^2^_1_=18.1, *P*<.001. A similar pattern emerged with regard to psychiatric medication: 62.8% of those who had never taken psychiatric medication would try the program, and 66.3% of those who had ever been prescribed psychiatric medication would try the program, χ^2^_1_=0.5, *P*=.48. Of those who would never consider using medication if needed, 57.8% would try our program, whereas 67.7% of those who would consider using medication would try our program, χ^2^_1_=5.5, *P*=.02.

### Predictors of Interest

In the first hierarchical linear regression predicting continuous attitude score, the blocks containing race, age, and marital status failed to improve model fit even marginally. Therefore, as planned, these blocks were removed from the subsequent analyses, and the second hierarchical regression included blocks for gender, SES, psychological symptoms, past treatment use, and hypothetical treatment use. At this stage, the following variables significantly predicted more positive attitude toward CMH (at the step in which they were entered): female gender, past use of therapy, and willingness to consider using therapy. Greater psychological symptoms marginally predicted positive attitude, and greater income was marginally associated with worse attitude. Full results of these initial analyses appear in Multimedia Appendix 2.

In a simultaneous regression, the aforementioned predictors with *P*<.10 were retained and all others were removed (Table 2). In this reduced model, the magnitude of estimated associations between attitude and gender, symptoms, and willingness to consider therapy were similar to their magnitudes in the previous model. Income became a significant predictor once education was removed, and past use of therapy became nonsignificant when entered simultaneously with willingness to consider therapy.

**Table 2:** Simultaneous regression predicting attitude toward the Crowdsourcing Mental Health program.

Predictor	*B*	SE *B*	95% CI *B*	*P* value
Female^a^	0.200	0.095	0.013 to 0.386	.04
Income^b^	−0.055	0.020	−0.095 to −0.016	.006
Brief Symptom Inventory	0.112	0.077	−0.039 to 0.263	.14
Ever used therapy	0.073	0.097	−0.118 to 0.263	.45
Would consider therapy	0.586	0.101	0.387 to 0.784	<.001

^a^Reference category is combined male and gender nonconforming respondents.

^b^Unit is income per household member^1/2^in US $10,000 increments.

In the first hierarchical logistic regression predicting whether respondents indicated willingness to participate in the program on the dichotomous item, the following blocks failed to marginally or significantly improve model fit: gender, marital status, and past treatment use. In the next regression, in which those blocks were removed, the block in which race or ethnicity dummy codes were entered marginally improved model fit, apparently driven by Hispanic respondents’ greater likelihood of indicating willingness to participate than non-Hispanic white respondents. All other blocks significantly improved model fit, and all predictors within those blocks were marginal or significant predictors of willingness to try the program except for willingness to consider taking psychiatric medication. See Multimedia Appendix 2 for detailed results of these analyses.

Therefore, for the simultaneous regression (Table 3), only willingness to consider medication was removed (the dummy codes for all race or ethnicity categories were retained so that the reference category would not change). In this analysis, individuals who were older or had higher income were less likely to indicate that they would try the program, and individuals who identified as Hispanic or who would be willing to consider using psychotherapy were more likely to state that they would try the program.

**Table 3:** Simultaneous regression predicting intention to try the Crowdsourcing Mental Health program.

Predictor	*B*	SE *B*	95% CI *B*	*P* value
Hispanic^a^	1.102	0.512	0.100 to 2.11	.03
Black^a^	0.221	0.356	−0.479 to 0.921	.53
Asian^a^	0.682	0.461	−0.224 to 1.587	.14
Native American^a^	−0.169	0.891	−1.918 to 1.581	.85
Other race^a^	−0.484	0.443	−1.354 to 0.386	.28
Age	−0.015	0.007	−0.029 to −0.001	.04
Income^b^	−0.087	0.041	−0.167 to −0.007	.03
Education	−0.123	0.066	−0.255 to 0.007	.06
Brief Symptom Inventory	0.292	0.165	−0.033 to 0.616	.08
Would consider therapy	0.936	0.194	0.556 to 1.317	<.001

^a^Reference category is non-Hispanic white.

^b^Unit is income per household member^1/2^in US $10,000 increments.

In the first hierarchical logistic regression predicting whether participants provided an email address to request more information, blocks including gender, race, symptoms, and hypothetical treatment use significantly improved model fit; no other blocks approached significance. In the second hierarchical logistic regression, female gender, symptoms, and openness to using psychotherapy all significantly predicted provision of an email address; Asian participants were significantly less likely to provide an email address than white participants. No other predictors approached significance. The full results of these regression analyses can be found in Multimedia Appendix 2.

In the simultaneous regression predicting email provision, willingness to consider medication and the dummy code for gender nonconforming were dropped (such that the reference category for gender was now non–female-identified rather than male). In this analysis, presented in Table 4, female gender, psychological symptoms, and openness to using therapy remained significant predictors.

**Table 4:** Simultaneous regression predicting provision of email address.

Predictor	*B*	SE *B*	95% CI *B*	*P* value
Female^a^	0.411	0.195	0.023 to 0.794	.04
Hispanic^b^	0.180	0.387	−0.581 to 0.939	.64
Black^b^	0.466	0.337	−0.196 to 1.128	.17
Asian^b^	−1.230	0.642	−2.491 to 0.031	.06
Native American^b^	1.207	0.933	−0.626 to 3.040	.20
Other race^b^	−0.093	0.473	−1.022 to 0.836	.84
Brief Symptom Inventory	0.471	0.149	0.179 to 0.764	.002
Would consider therapy	0.929	0.217	0.503 to 1.355	<.001

^a^Reference category is combined male and gender nonconforming respondents.

^b^Reference category is non-Hispanic white.

To summarize the significant results from the final models, female gender predicted favorable attitudes and email provision; Hispanic ethnicity predicted intention to try CMH; older age was associated with lower endorsement of willingness to try CMH; lower income predicted favorable attitudes and intention to try CMH; higher psychological distress was associated with email provision; and willingness to use psychotherapy predicted all 3 dependent variables.

### Preferred Partners

Table 5 lists the proportion of participants who ranked each potential partner first and second and the results of pairwise comparisons of the ranks of each partner type. Most individuals preferred a romantic partner as a first choice, with a close friend as a common second choice. A notable minority of respondents ranked a stranger as their ideal peer counselor.

**Table 5:** Most preferred peers.

Partner type	Ranked 1, n (%) (N=588)	Ranked 2, n (%) (N=588)	Mean rank^a^
Romantic partner or spouse	339 (57.7)	88 (15.0)	1.96
Close friend	102 (17.3)	232 (39.5)	2.39
Family member	59 (10.0)	161 (27.4)	3.01
Acquaintance	25 (4.3)	70 (11.9)	3.55
Stranger	63 (10.7)	37 (6.3)	4.08

^a^All pairwise comparisons between ranks were significantly different, *P*<.005.

### Desired Counseling Skills

In order to identify the skills that consumers believe would be most valuable to learn in a peer support program, the survey listed counseling skills and asked respondents to rate how important it was to them that their peer learn that skill, as well as how important it was that they themselves learn that skill, on a 1 to 7 Likert scale from “not at all important” to “extremely important.” Table 6 provides the proportion of respondents rating each skill as “very” or “extremely” important to learn, as well as the results of within-subjects *t* tests of the difference in importance between one’s peer learning each skill and learning it oneself.

**Table 6:** Importance of learning various peer counseling skills.

Peer counseling skills	Rated skill “very” or “extremely” important, n (%) (N=592)		Mean difference in ratings	*t*_591_	*P* value
	Want peer to learn	Want self to learn			
How to genuinely listen	474 (80.6)	366 (61.9)	0.58	11.06	<.001
How to pay attention	439 (74.6)	351 (59.3)	0.51	9.37	<.001
How to show understanding	416 (70.7)	377 (63.7)	0.23	4.56	<.001
How to empathize	403 (68.5)	366 (61.8)	0.28	5.28	<.001
How to avoid being judgmental	397 (67.5)	357 (60.3)	0.28	4.87	<.001
How to be compassionate	387 (65.9)	378 (63.9)	0.14	2.94	.003
How to comfort the other person	357 (60.7)	363 (64.7)	−0.05	−0.94	.35
How to help think through decisions	348 (59.1)	371 (62.6)	−0.13	−2.44	.02
How to help solve practical problems	310 (52.7)	334 (56.5)	−0.10	−1.86	.06
How to give advice	308 (52.4)	345 (58.3)	−0.11	−2.03	.04

The most highly prized skills involved simply listening attentively and taking an understanding, nonjudgmental stance. Generally, respondents considered it more important for their peer to learn each skill than themselves. However, this pattern was weaker or even reversed for skills related to intervening to resolve the other person’s distress, for example, by solving problems or comforting the other person. For those “intervention-like” skills, participants wanted to learn to deliver the skills to their peers more than they wanted their peers to learn the skills.

### Access Channels

To determine the method of access that would reach the most consumers, we provided respondents with a choice of 6 ways to access the peer counseling training course and intervention: through a stand-alone website, through a social networking website such as Facebook, as a mobile “app,” as a program offered through a doctor’s office or other health care provider, as a program offered through one’s workplace or school, or as a program offered through a community center such as a public library or the YMCA (Young Men’s Christian Association). Respondents were asked to indicate the way they would be most likely to access the program by rank-ordering the options. They also rated each option on several 6-point semantic differential scales: appealing-unappealing, trustworthy-untrustworthy, convenient-inconvenient, and easy to use–hard to use.

The most popular access choice by far was stand-alone website, with 51.2% (303/592) of respondents ranking it as their most likely option, with doctor’s office and mobile app following (see Table 7). On the basis of the semantic differential ratings (which are summarized in Table 8), respondents viewed a website as most appealing, convenient, and easy to use and second in trustworthiness. Accessing peer counseling through a doctor’s office or health care provider was seen as most trustworthy but least convenient; in contrast, mobile apps were rated as relatively convenient but less trustworthy and harder to use. Community centers were moderately trustworthy but less convenient, and work or school was moderately convenient and trustworthy. Social networking sites were the least popular option, rated low in appeal and trustworthiness, although moderate in convenience and ease of use.

**Table 7:** Most likely method for accessing peer counseling.

Method of access	Ranked 1, n (%) (N=588)	Ranked 2, n (%) (N=588)	Mean rank^a^
Website	301 (51.2)	111 (18.9)	2.12
Doctor’s office	86 (14.6)	95 (16.2)	3.44^b^
Mobile app	68 (11.6)	138 (23.5)	3.66^b,c^
Community center	46 (7.8)	94 (16.0)	3.69^c^
Work or school	49 (8.3)	72 (12.2)	3.87^c^
Social networking site	38 (6.5)	78 (13.3)	4.22

^a^Lower mean ranks indicate greater preference.

^b^These options did not differ significantly when compared, Wilcoxon signed rank test *P*<.0033.

^c^These options did not differ significantly, Wilcoxon signed rank test *P*<.0033.

**Table 8:** Access methods’ mean ranks on each attribute.

Method of access	Mean rank^a^			
	Appealing	Trustworthy	Convenient	Easy to use
Website	2.57	3.03^b^	2.66	1.79
Doctor’s office	3.46^b^	2.55	4.18^c^	-
Mobile app	3.85^c^	4.47^c^	3.27^b^	2.15
Community center	3.44^b^	3.14^b^	4.04^c^	-
Work or school	3.74^c^	3.32	3.54^b^	-
Social networking site	3.93^c^	4.49^c^	3.30^b^	2.06

^a^Attributes not assessed for a particular access method are marked by a dash. Lower mean ranks indicate greater preference.

^b^These options did not differ significantly when compared, Wilcoxon signed rank test *P*<.0033.

^c^These options did not differ significantly, Wilcoxon signed rank test *P*<.0033.

## Discussion

### Overall Interest in the Program

The purpose of this investigation was to assess consumer interest in a Web-based self-help and peer support mental health intervention, to determine demographic and clinical predictors of interest in the intervention, and to evaluate consumer preferences for specific features of Web-disseminated peer support interventions.

Respondents expressed fairly high interest in the CMH program: 63.9% verbally communicated that they would try the program, and 32.6% showed behavioral evidence of interest by offering their email addresses to request more information. More than half of respondents who indicated that they would never consider seeking psychotherapy or psychiatric medication were willing to try the CMH program. This proportion was significantly lower than it was among those who were willing to access these traditional mental health services, which indicates that programs like CMH are not a panacea; they cannot reach every person who is “left out” by traditional services. However, it appears that peer counseling still appeals to a substantive portion of that population.

### Predictors of Interest

Several demographic and clinical characteristics were associated with 3 different indicators of interest in the CMH program: a multi-item measure of the program’s appeal (continuous), endorsement of intention to try the program (dichotomous), and provision of an email address in order to receive information about the program (dichotomous). Female gender, lower income, and openness to using psychotherapy were associated with positive attitude toward CMH while controlling for other variables. Younger age, Hispanic ethnicity, lower income, and openness to using psychotherapy predicted willingness to try the program while controlling for other variables. Female gender, higher symptom distress, and openness to using therapy were associated with provision of an email address while controlling for other variables.

#### Gender

We predicted that females would be more interested in the CMH program than males in light of their greater willingness to seek both eHealth and traditional mental health services [28-30], which may be related to gender differences in rates of mental illnesses as well as to lower perceived stigma among women [70]. However, the association between gender and interest remained when controlling for hypothetical use of professional mental health care, suggesting that there are other reasons women find CMH more appealing than men do. Perhaps the prospect of reciprocal social support is attractive to women to an even greater degree than professional help. According to Taylor and colleagues’ [71] “tend and befriend” theory, women are evolutionarily prepared to respond to stress with affiliative behaviors and building of social networks. Women also place higher demands upon friendships than men, expecting more reciprocity and intimacy [72]. Therefore, women may value CMH and other peer support programs’ efficacy for meeting the needs of enhancing relationships and social resources.

The association between gender and interest in the program suggests that CMH and other peer support programs do not completely avoid the barriers men face to seeking mental health care. This makes sense when one considers the demands of the male gender role. CMH has the potential to reduce stigma because users do not have to identify as mentally ill; however, it still requires that users seek support from one another and disclose their personal experiences, thoughts, and feelings. Such support-seeking and vulnerability violate cultural scripts of traditional masculinity [70,73]. However, the associations between gender and interest in the program were small, suggesting that many men were still interested in CMH.

#### Income

The association between interest in CMH and lower income is particularly intriguing in light of previous research showing that people with lower income have more negative attitudes toward seeking professional psychological help (eg, [29]). It may be that people with lower income find professional care less appealing and instead prefer to utilize nonprofessional sources of help. A national survey found that low income was associated with seeking support for health problems from one’s personal social network rather than professionals [74]. Thus, leveraging existing interpersonal relationships for delivery of health care may be an ideal way to reach low-income individuals.

#### Age

Older respondents were less likely to endorse willingness to try the CMH program; however, age did not predict participants’ attitudes toward the program or their likelihood of requesting more information via email. Older age is associated with lower technology adoption [75], including lower use of eHealth [28]. Therefore, dissemination of Web-based interventions to older individuals might be facilitated by ensuring that interfaces are intuitive to users of all ages and by promoting programs as user-friendly. It may also be helpful to supplement Web interventions with face-to-face support or to secure endorsement from trusted individuals (eg, health care providers; [76]).

#### Race or Ethnicity

Respondents who identified as Hispanic were more likely to indicate that they would try the program than non-Hispanic white respondents. (Although the association between Hispanic ethnicity and attitudes did not reach significance, it was in the same direction.) This result requires replication, given that it was not consistent across outcome measures and only 33 respondents selected this ethnic category. Furthermore, there is a great deal of cultural heterogeneity among Hispanic Americans. However, one can speculate that Hispanic respondents’ greater willingness to use the CMH program may be related to the value of *familismo*, which features prominently in research on Hispanic cultural psychology (eg, [77]). Hispanic individuals’ strong family support systems and desire to keep family matters private have been given as reasons for limited utilization of professional mental health care among this group [78]. In contrast to traditional mental health services, programs like CMH that train individuals to improve their mental health through existing social ties may be a particularly culturally acceptable strategy for reaching individuals high in *familismo*.

No other racial or ethnic identity group differed from non-Hispanic whites in level of interest in the CMH program. For many racial or ethnic minorities, stigma around mental illness is higher than among white individuals [79], and there may be distrust of health care providers [80], which partially drives negative attitudes toward traditional mental health services among minorities [81]. Perhaps by eliminating the barrier of stigma by promoting mental health without requiring that users receive a label of “disordered” or “ill,” and by enabling users to receive support from those they trust, CMH and programs like it can decrease the racial mental health disparities that persist in the United States [82].

#### Clinical Characteristics

Openness to using psychotherapy was the most consistent predictor of interest in CMH across the 3 outcome variables, even when controlling for all demographic and clinical characteristics. This suggests that some of the same, unmeasured factors drive willingness to access therapy and interest in CMH. On the other hand, openness to using therapy accounted for less than 6% of the variance in attitudes toward CMH, and many individuals who indicated they would not use psychotherapy endorsed willingness to try CMH. This suggests that the shared features of professional psychotherapy and Web-based self-help and peer support programs account for *some* common level of appeal but that, for the most part, these traditional and nontraditional interventions fill *separate* ecological niches in the mental health care system. Rather than focusing on disseminating a handful of traditional strategies for treating mental health problems, researchers and policy makers should consider investing effort in developing a diverse portfolio of tools in order to maximize reach and impact on public mental health.

In the hierarchical models, symptom distress was significantly or marginally associated with each index of interest such that people with more symptoms expressed more interest in the CMH program. Distress remained a significant predictor of requesting more information via email when controlling for other variables. The general positive association between distress and interest is heartening—CMH and similar programs will appeal most to those who need them most.

### Preferred Partners

When asked whom they would prefer as a partner in the CMH program, respondents generally favored romantic partners or close friends, although a notable minority of respondents preferred to work with a stranger. These findings on selecting a peer suggest that it is important for anyone creating a peer support intervention to recognize that, although such interventions have typically paired 2 strangers, most people would rather work with someone whom they already know, preferably someone with whom they are close. At the same time, some individuals—perhaps those with narrower social circles or conflictual close relationships—find disclosing to a stranger attractive. Peer support’s reach might be optimized by designing interventions that are flexible enough to allow for a variety of people to act as peers and by building in processes for introducing strangers who do not want to work with friends or family.

Additionally, the popularity of a romantic partner as a peer counselor may also imply that many people are interested in strengthening their romantic relationships, a possibility corroborated by many comments in an open-ended section of the survey about desiring to improve communication with spouses. Web-based interventions for couples or spouses may present an additional opportunity to improve public mental health, given the evidence that interventions for couples can improve individual mental health in addition to relationship functioning [83].

### Desired Counseling Skills

When presented with a list of counseling skills that they or their peers could learn, respondents expressed the greatest desire that their peers learn skills that involved attentive, nonjudgmental listening. Respondents generally considered it more important for their peer to learn each skill than to learn that skill themselves, perhaps because people are more invested in receiving quality support than providing it or because people tend to overestimate their own abilities [84]. This disparity was especially apparent for active listening skills, but this pattern was weaker or even reversed for skills related to resolving the other person’s distress. In other words, respondents wanted to learn to “fix” stressors for their peers but just wanted their peers to learn to listen empathically.

These findings converge with several lines of work that illuminate helpful and unhelpful ways to react to others’ distress. Well-intentioned peers and loved ones often respond to disclosures of distress by trying to eliminate the stressful stimulus (eg, by solving problems or giving advice) or by trying to change the discloser’s emotional response (eg, by reframing the stressor in a positive light or minimizing its gravity). Yet the recipients of such “support” tend to regard it as unhelpful or even disturbing [44,59-61]. The reasons why attempts to change the stressor or the emotional response could be harmful are not yet thoroughly investigated, but such support attempts may be perceived as judgmental, make recipients feel misunderstood and more alone, or interfere with self-verification strivings (ie, a motivation to receive feedback that confirms self-conceptions) [85]. In contrast, social support recipients tend to appreciate loved ones simply being present, expressing empathy, being accepting, or validating their feelings [44,59-61].

On the basis of this literature and the corroborating results of our survey, those who hope to create new peer support interventions should consider incorporating training in attentive, nonjudgmental listening and reflection skills, as well as education about the potential dangers of common “helping” behaviors such as giving reassurance and advice. However, one must recognize that what support seekers *prefer* may not necessarily correspond with what is *effective*. Purely “supportive” psychotherapies are not as effective as those that contain more “active” ingredients [86]. If the creators of peer support training programs do plan to teach peers to administer more active problem-solving or extrinsic emotion regulation, they should make sure support providers are thoroughly trained and monitored to do so sensitively, lest the support be regarded as unhelpful or damage the relationship.

### Access Channels

Selecting from 6 options for learning about and accessing the Web-based training course, the overwhelming majority of respondents indicated that they would be most likely to use the program if it took the form of a stand-alone website. A notable minority indicated that a health care provider’s office or a mobile app would be the ideal point of access. A website and a mobile app were regarded as convenient, and health care providers were regarded as highly trustworthy but inconvenient. These results suggest that a training course is likely to have the greatest reach if offered via a stand-alone website. However, offering a mobile version of the course, or disseminating peer counseling resources through existing health care providers, could expand the population served, given that 11.6% and 14.6% of respondents, respectively, ranked those access options as the most likely way to reach them. Perhaps developers of Web-disseminated self-help or peer support courses could capitalize on the trustworthiness of health care providers by using doctors’ offices as a first point of contact but could increase convenience by enabling users to access the course and meet with peers at any location or on any device of their choosing.

### Limitations and Future Directions

The chief limitation of this study was the use of a nonprobability sample. Although diverse, MTurk users are not representative of the population, and they may be somewhat more interested in this program than the general public because of their comfort with using technology.

An additional limitation of this study is that the findings regarding interest in the program apply to the specific description of the program used in the survey. Interest may have differed if the program were presented in more detail or with other emphases. Furthermore, Web-disseminated self-help or peer support programs that include different features may engender different levels of consumer interest than the CMH program.

Future research on such programs could expand upon the foundation laid here in a variety of ways. This exploratory study established that interest exists; however, it did not explain *why* respondents were interested. It would be helpful to know which elements of this Web-based self-help and peer support training drive consumers’ attraction and which elements are neutral or even unappealing. Additionally, although we found some differences in interest among demographic groups, we could only speculate on reasons for the associations between interest and demographic characteristics. Further investigations could probe the underlying causes of these associations in order to inform how such programs could be more responsive to the values and life circumstances of diverse individuals. Finally, it may be especially fruitful to combine these two research threads and examine interactions between demographics and preferences for specific elements of such programs, thus identifying elements that are particularly desirable to certain demographic groups so that interventions can be tailored to their needs.

### Conclusions

The results of this study substantiate the potential for CMH to fill gaps in mental health care. Diverse consumers would be interested in such an intervention, including those who are not already accessing services. Because they minimize stigma and utilize existing social support systems, reciprocal peer support interventions may be especially attractive to some groups who are underserved by professional mental health services.

It appears that CMH’s appeal can be enhanced by allowing a variety of options for peer counselors and by teaching active listening skills. It also seems that the course will reach the greatest number of users if accessed via a website, although access through mobile apps or through health care providers’ offices may also be useful options.

We hope that the results of these surveys will persuade readers of the potential utility of Web-disseminated self-help and peer support programs and will inform the creation and dissemination of other programs.

## Appendix

Multimedia Appendix 1 The description of the program presented in the survey.

Multimedia Appendix 2 The results of the hierarchical linear and logistic regressions.

## Abbreviations

BSI: Brief Symptom Inventory
CMH: Crowdsourcing Mental Health
IP: Internet protocol
MOOC: massive open online course
MTurk: Mechanical Turk
SES: socioeconomic status
YMCA: Young Men’s Christian Association
