# Supplementary material for: A Web-Disseminated Self-Help and Peer Support Program Could Fill Gaps in Mental Health Care: Lessons From a Consumer Survey
Source: JMIR Ment Health. 2017 Jan 19;4(1):e5. doi: 10.2196/mental.4751 (PMC5290297; doi:10.2196/mental.4751)
Supplement: Multimedia Appendix 1 [file mental_v4i1e5_app1.pdf]

Please CAREFULLY read the description of the program we are developing. You will be asked questions to make sure you understand what you read.

We are developing a new program that is intended to increase people's well-being.

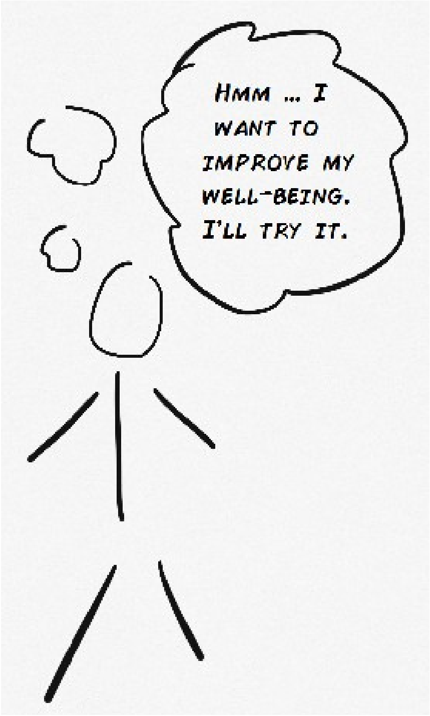

First, people who are interested in participating will choose someone to be their partner in the program. This partner will usually be someone they already know.

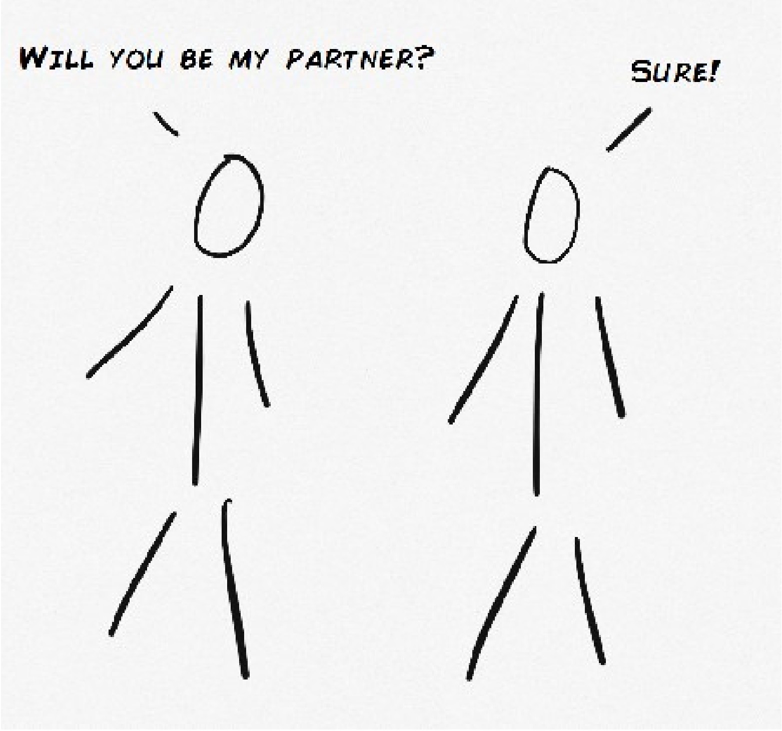

Then, both people will take a training course where they learn skills for talking and listening to each other constructively and helpfully. The training will be offered over the Internet.

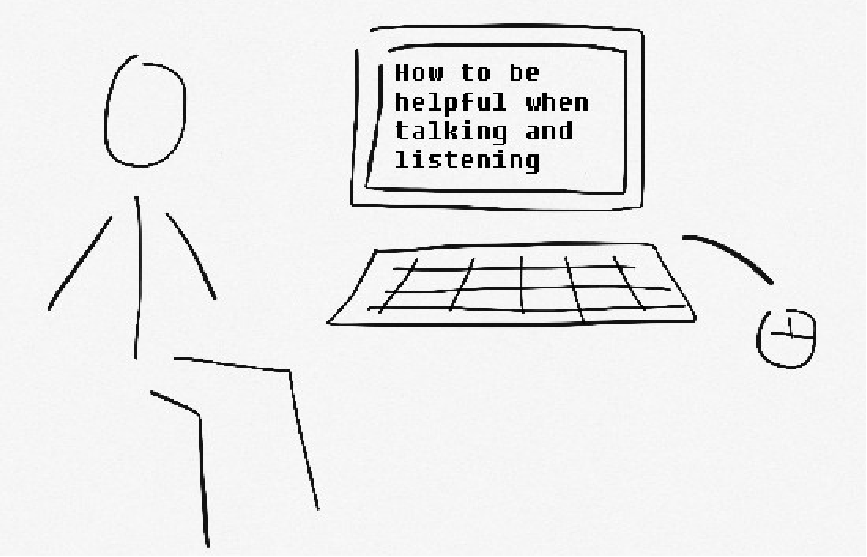

After both people complete the training, they will meet regularly to talk to each other. First, one person will get a chance to talk while the other one listens and responds helpfully. Then the roles will switch: the second person will talk while the first person listens and responds helpfully.

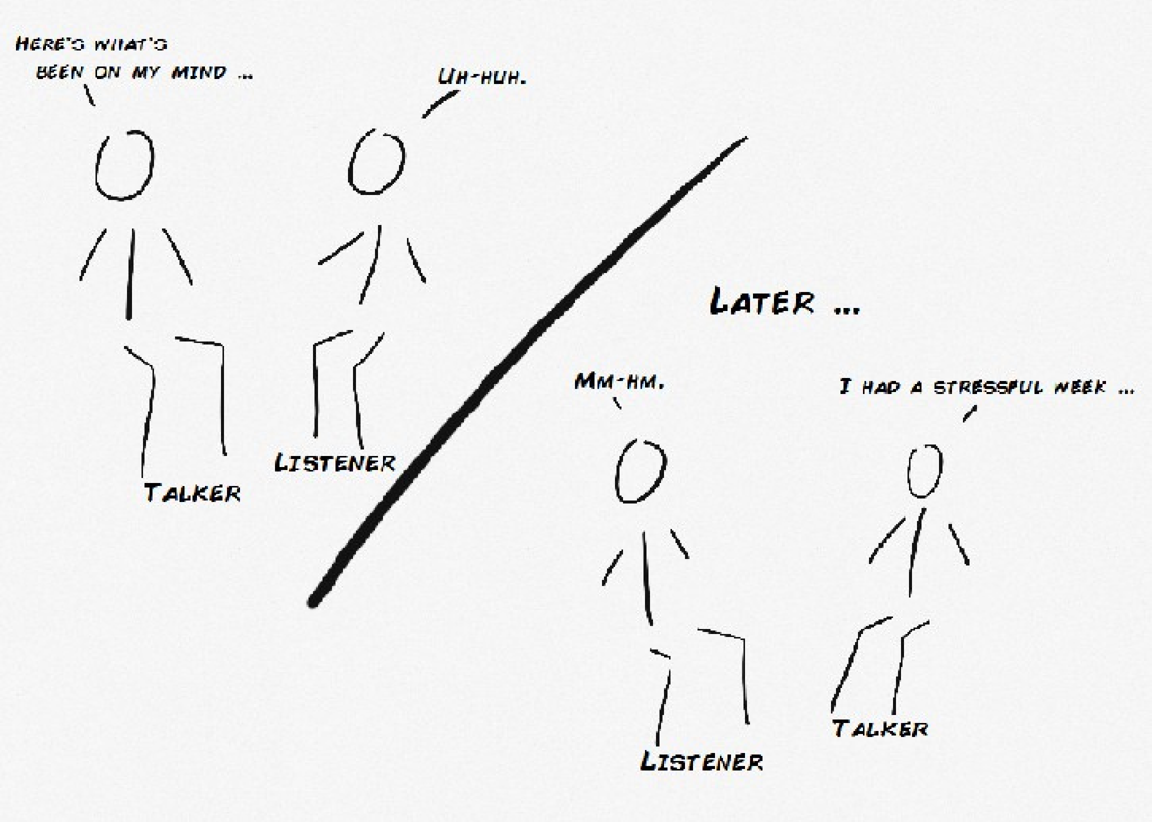

We expect this program to improve participants' lives in a variety of ways. We hope that participants will better manage their stress and negative emotions, will get closer to their partners, will figure out how to solve problems in their lives, and will understand themselves better. We also hope that as a result of these benefits, participants will be able to deal with minor mental health problems without having to see a professional.
