# Supplementary material for: A Web-Disseminated Self-Help and Peer Support Program Could Fill Gaps in Mental Health Care: Lessons From a Consumer Survey
Source: JMIR Ment Health. 2017 Jan 19;4(1):e5. doi: 10.2196/mental.4751 (PMC5290297; doi:10.2196/mental.4751)
Supplement: Multimedia Appendix 2 [file mental_v4i1e5_app2.pdf]

Appendix Table 1. Regression predicting attitude score from all variables

Step	Predictor	*B*	SE *B*	*P*	R^2	ΔR^2	D_m[a]	*P*
1: Gender[b]	Female	0.271	0.098	.006	.0129		3.84	.02
	Transgender	0.116	0.257	.65				
2: Race/ethnicity[c]	Hispanic	0.356	0.205	.08	.0245	.0116	1.39	.22
	Black	0.261	0.180	.15				
	Asian	0.201	0.225	.37				
	Native American	0.591	0.467	.21				
	Other	-0.083	0.238	.73				
3: Age	Age	-0.004	0.004	.34	.0260		0.93	.34
4: Socioeconomic status	Income[d]	-0.041	0.022	.06	.0372	.0110	3.39	.03
	Education	-0.039	0.035	.263				
5: Marital status[e]	Single	-0.095	0.104	.36	.0388	.0016	0.32	.81
	Separated/divorced	-0.080	0.180	.66				
	Widowed	-0.126	0.359	.73				
6: Symptoms	Brief Symptom Inventory	0.142	0.081	.08	.0439	.0051	3.0	.08
7: Past treatment	Ever used therapy	0.231	0.116	.047	.0553	.0114	3.47	.03
	Ever used medication	0.053	0.126	.67				
8. Hypothetical treatment	Would consider therapy	0.581	0.117	< .001	.1139	.0586	18.84	< .001
	Would consider medication	0.086	0.111	.44				

[a]D_m is the model comparison statistic described by Meng and Rubin (1992).
[b]Reference category is male.
[c]Reference category is non-Hispanic White.
[d]Unit is income per household member$^{1/2}$ in $10,000 increments.
[e]Reference category is married/cohabiting.

Appendix Table 2. Regression predicting attitude score with blocks omitted that did not improve fit

Step	Predictor	*B*	SE *B*	*P*	R^2	ΔR^2	D_m[a]	*P*
1: Gender[b]	Female	0.271	0.098	.006	.0129		3.84	.02
	Transgender	0.116	0.257	.65				
2: Socioeconomic status	Income[c]	-0.042	0.022	.06	.0255	.0126	4.14	.02
	Education	-0.044	0.034	.20				
3: Symptoms	Brief Symptom Inventory	0.143	0.079	.07	.0310	.0055	3.28	.07
4: Past treatment	Ever used therapy	0.235	0.114	.04	.0406	.0096	2.89	.06
	Ever used medication	-0.001	0.124	.99				
5. Hypothetical treatment	Would consider therapy	0.551	0.116	< .001	.0945		17.30	< .001
	Would consider medication	0.080	0.111	.47				

[a]D_m is the model comparison statistic described by Meng and Rubin (1992).
[b]Reference category is male.
[c]Unit is income per household member$^{1/2}$ in $10,000 increments.

Appendix Table 3. Logistic regression predicting intention to try CMH from all variables

Step	Predictor	*B*	SE *B*	*P*	D_m[a]	*P*
1: Gender[b]	Female	0.236	0.178	.18	0.90	.41
	Transgender	-0.008	0.462	.99		
2: Race/ethnicity[c]	Hispanic	1.232	0.496	.01	2.16	.06
	Black	0.237	0.339	.48		
	Asian	0.376	0.433	.39		
	Native American	0.184	0.873	.83		
	Other	-0.533	0.421	.21		
3: Age	Age	-0.015	0.007	.03	4.74	.03
4: Socioeconomic status	Income[d]	-0.075	0.040	.06	4.91	.007
	Education	-0.119	0.065	.07		
5: Marital status[e]	Single	-0.168	0.196	.39	0.28	.84
	Separated/divorced	-0.124	0.335	.71		
	Widowed	0.103	0.6612	.88		
6: Symptoms	Brief Symptom Inventory	0.378	0.168	.02	5.402	.02
7: Past treatment	Ever used therapy	0.067	0.222	.76	0.13	.88

	Ever used medication	0.046	0.241	.84		
8. Hypothetical treatment	Would consider therapy	0.951	0.233	< .001	12.07	< .001
	Would consider medication	0.075	0.223	.74		

[a]D_m is the model comparison statistic described by Meng and Rubin (1992).
[b]Reference category is male.
[c]Reference category is non-Hispanic White.
[d]Unit is income per household member$^{1/2}$ in $10,000 increments.
[e]Reference category is married/cohabiting.

Appendix Table 4. Logistic regression predicting intention to try CMH with blocks omitted that did not improve fit

Step	Predictor	*B*	SE *B*	*P*	D_m[a]	*P*
1: Race/ethnicity[b]	Hispanic	1.224	0.495	.01	2.11	.06
	Black	0.263	0.338	.44		
	Asian	0.366	0.846	.40		
	Native American	0.194	0.871	.82		
	Other	-0.499	0.420	.23		
2: Age	Age	-0.014	0.007	.03	4.54	.03
3: Socioeconomic status	Income[c]	-0.077	0.040	.06	4.80	.008
	Education	-0.112	0.064	.08		
4: Symptoms	Brief Symptom Inventory	0.349	0.165	.03	4.76	.03
5. Hypothetical treatment	Would consider therapy	0.915	0.227	< .001	11.89	< .001
	Would consider medication	0.038	0.218	.86		

[a]D_m is the model comparison statistic described by Meng and Rubin (1992).
[b]Reference category is non-Hispanic White.
[c]Unit is income per household member$^{1/2}$ in $10,000 increments.

Appendix Table 5. Logistic regression predicting provision of email from all variables

Step	Predictor	*B*	SE *B*	*P*	D_m[a]	*P*
1: Gender[b]	Female	0.461	0. 189	.02	3.24	.04
	Transgender	-0.181	0.564	.75		
2: Race/ethnicity[c]	Hispanic	0.176	0.378	.642	2.22	.05
	Black	0.315	0.324	.33		
	Asian	-1.35	0.622	.03		
	Native American	1.42	0.877	.11		
	Other	-0.219	0.462	.64		
3: Age	Age	0.005	0.007	.44	0.60	.44
4: Socioeconomic status	Income[d]	-0.036	0.043	.40	0.79	.45
	Education	-0.042	0.066	.53		
5: Marital status[e]	Single	-0.281	0.201	.16	0.69	.56
	Separated/divorced	-0.008	0.332	.98		
	Widowed	-0.154	0.666	.82		
6: Symptoms	Brief Symptom Inventory	0.562	0.153	< .001	13.46	< .001
7: Past treatment	Ever used therapy	0.324	0.223	.15	1.93	.14
	Ever used medication	0.088	0.239	.71		
8. Hypothetical treatment	Would consider therapy	0.905	0.253	< .001	8.69	< .001
	Would consider medication	0.008	0.229	.971		

[a]D_m is the model comparison statistic described by Meng and Rubin (1992).
[b]Reference category is male.
[c]Reference category is non-Hispanic White.
[d]Unit is income per household member$^{1/2}$ in $10,000 increments.
[e]Reference category is married/cohabiting.

Appendix Table 6. Logistic regression predicting provision of email with blocks omitted that did not improve fit

Step	Predictor	*B*	SE *B*	*P*	D_m[a]	*P*
1: Gender[b]	Female	0.461	0. 189	.02	3.24	.04
	Transgender	-0.181	0.564	.75		
2: Race/ethnicity[c]	Hispanic	0.176	0.378	.64	2.22	.05

	Black	0.315	0.324	.33		
	Asian	-1.350	0.623	.03		
	Native American	1.415	0.877	.11		
	Other	-0.219	0.462	.64		
3: Symptoms	Brief Symptom Inventory	0.487	0.146	< .001	11.02	< .001
4. Hypothetical treatment	Would consider therapy	0.911	0.247	< .001	10.22	< .001
	Would consider medication	0.055	0.221	.80		

[a] D_m is the model comparison statistic described by Meng and Rubin (1992).
[b] Reference category is male.
[c] Reference category is non-Hispanic White.
